# Application of Antimicrobial Polymers in the Development of Dental Resin Composite

**DOI:** 10.3390/molecules25204738

**Published:** 2020-10-15

**Authors:** Jing Xue, Jing Wang, Daoshuo Feng, Haofei Huang, Ming Wang

**Affiliations:** School of Chemistry and Chemical Engineering, Shandong University of Technology, 266 Xincun Road, Zibo 255000, China; xuejingsdut@163.com (J.X.); fengdaoshuosdut@163.com (D.F.); 1982hhf@163.com (H.H.); wangmingmw@sdut.edu.cn (M.W.)

**Keywords:** dental resin composites, mechanical properties, antimicrobial properties, antimicrobial monomers, antimicrobial polymers

## Abstract

Dental resin composites have been widely used in a variety of direct and indirect dental restorations due to their aesthetic properties compared to amalgams and similar metals. Despite the fact that dental resin composites can contribute similar mechanical properties, they are more likely to have microbial accumulations leading to secondary caries. Therefore, the effective and long-lasting antimicrobial properties of dental resin composites are of great significance to their clinical applications. The approaches of ascribing antimicrobial properties to the resin composites may be divided into two types: The filler-type and the resin-type. In this review, the resin-type approaches were highlighted. Focusing on the antimicrobial polymers used in dental resin composites, their chemical structures, mechanical properties, antimicrobial effectiveness, releasing profile, and biocompatibility were included, and challenges, as well as future perspectives, were also discussed.

## 1. Introduction

Dental resin composites used for dental restorations, such as dental adhesive and dental filling composites, are composed of resin matrix and fillers, which can be self-cure or light-cure based on clinical requirements. Compared to metal restorations, bacterial accumulations or biofilm growth leading to secondary caries is a major drawback of resin composite restorations [1,2]. Therefore, ascribing antimicrobial properties to dental resin composites is of great interest for both academic purposes and clinical applications [3,4].

**Figure 1:**
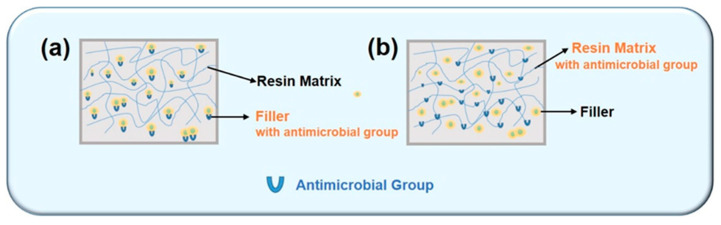
Two types of antimicrobial dental resin composite: (**a**) Filler-type and (**b**) resin-type.

Strategies for developing antimicrobial dental resin composites can be categorized into two types: Resin-type and filler-type (Figure 1). As for the filler-type, previous works mainly focused on the functionality of filler surfaces and incorporation of antimicrobial agents. The filler-type strategies usually correspond to the burst release of antimicrobial components, which is prone to occur, leading to a fast loss of antimicrobial effectiveness. From this point, resin-type strategies have the advantages of minimizing antimicrobial components releasing through covalent bonding, thus maintaining long-term antimicrobial properties. As dental resin composite is provided in an unpolymerized form in dental clinics for spreading or shaping purposes, and is either self-cured or light-cured upon polymerization, antimicrobial polymers in dental resin are in the form of antimicrobial monomers at the very beginning. The antimicrobial monomers are typically obtained by grafting biocide moieties onto methacrylate monomers to make it bear antimicrobial activities [5]. The antimicrobial monomers and several other monomers, typically methacrylates, are formulated to compose the resin matrix, and are co-polymerized to produce the final composite. Dental composites with antimicrobial resins are considered a new generation of bioactive dental restorative materials [6]. In this review, the development of antimicrobial monomers incorporated into dental resins was covered, and their antimicrobial properties before and after polymerization were focused. Furthermore, mechanical properties of dental resin composite with antimicrobial properties were included. Biocompatibility of the modified dental resin composites was also discussed based on the requirements for clinical applications.

## 2. Antimicrobial Monomers in Dental Resin

### 2.1. Methacryloyloxydodecylpyridinium Bromide (MDPB)

**Figure 2:**
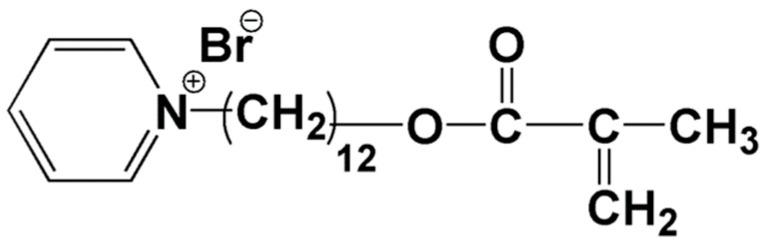
Chemical structure of methacryloyloxydodecylpyridinium bromide (MDPB).

Methacryloyloxydodecylpyridinium bromide (MDPB) (Figure 2) is one of the most important monomers for the development of antimicrobial dental resin composite, which was synthesized on the basis of hydroxydodecylpyridinium bromide, an antimicrobial agent [7]. By adding a methacryloyl group, MDPB was ascribed both antimicrobial properties and polymerizable abilities. When formulated into a dental filling composite composed of 2,2-bis[4-(3-methacryloxy-2-hydroxypropoxy) phenylpropane (Bis-GMA), triethyleneglycoldimethacrylate (TEGDMA) and dimethylaminoethylmethacrylate (DMAEMA) at the weight percent of 0.1% and 0.2%, respectively, MDPB could successfully copolymerize with the typical methacrylates to produce nonreleasing antibacterial effects. Except for dental filling composite, MDPB was also applied into dental adhesion systems. With the practice of adding MDPB to a proprietary self-etching primer [8], the commercial dental adhesive product of Clearfil Protect Bond™ (Kuraray Co. Ltd., Japan) could be formulated with MDPB to produce both in vitro and in vivo antimicrobial results [9].

### 2.2. 2-Methacryloyloxyethyl Phosphorylcholine (MPC)

**Figure 3:**
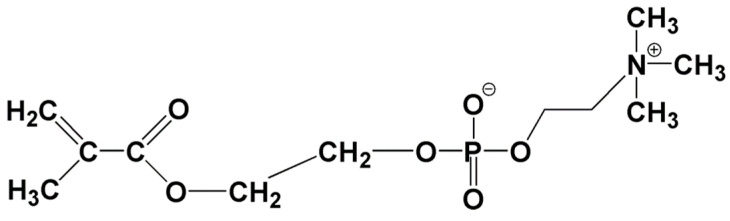
Chemical structure of 2-methacryloyloxyethyl phosphorylcholine (MPC).

Many cell types, such as erythrocyte cells and some bacteria, have a lipid bilayer membrane with asymmetric lipid composition [10], which have provided hints for the development of chemical entities used in biological systems. As for the lipid bilayer membrane, the inner layer is mainly constituted of negatively charged phospholipids like phosphatidylserine, while the outer layer contains a large proportion of zwitterionic lipid phosphatidylcholine [10]. The preparation of phosphorylcholine (PC) analogs paved the way to the synthesis of 2-methacryloyloxyethyl phosphorylcholine (MPC) (Figure 3) [11]. MPC could be obtained by coupling 2-hydroxyethylmethacrylate with 2-chloro-2-oxo-1,3,2-dioxaphospholane followed by the ring-opening of the intermediate alkoxyphospholane with trimethylamine [12]. The introduction of a methacryloyl group in MPC made it possible for polymerization reactions, and the PC headgroup is capable for bioactive purposes. Zhang et al. [13] incorporated MPC and a quaternary ammonium dimethylaminohexadecyl methacrylate, DMAHDM, into dental composite, and observed an enhanced inhibition effect on the biofilm growth and lactic acid production. It was suggested that DMAHDM played a major role in the antimicrobial effect, while MPC assisted this process through a protein-repellent, producing a synergistic result. The quaternary ammonium methacrylates (QAMs) will be discussed more in the following sections.

### 2.3. Quaternary Ammonium Methacrylates (QAMs)

**Figure 4:**
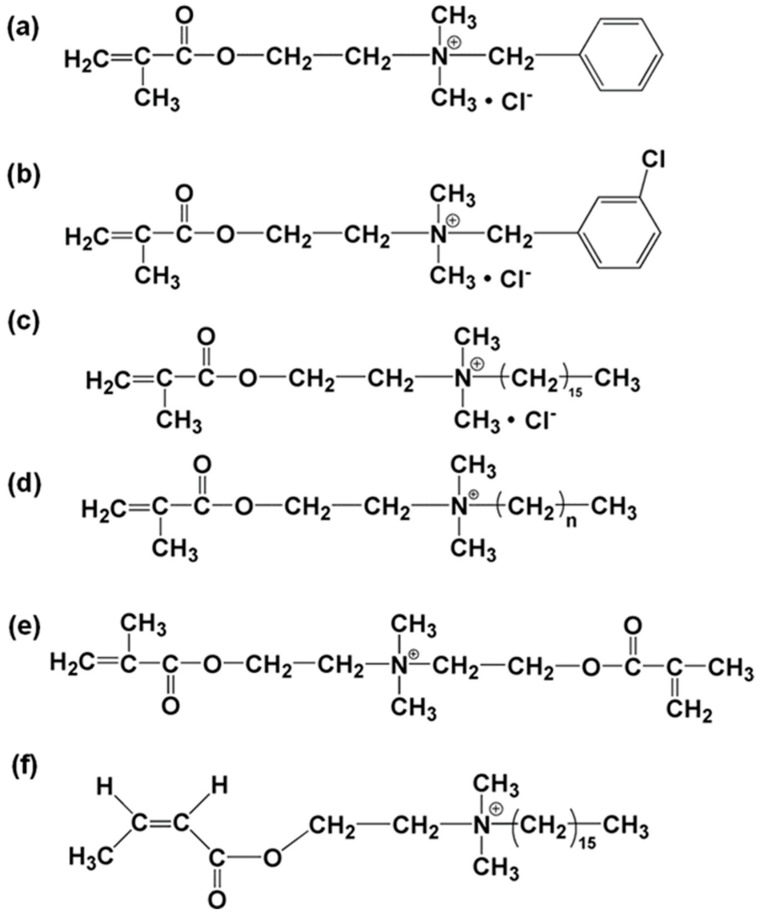
Chemical structure of quaternary ammonium methacrylates (QAMs) (quaternary ammonium salts (QASs)) monomers: (**a**) DMAE-BC; (**b**) DMAE-m-CBC; (**c**) DMAE-CB; (**d**) DMA-DMADDM (n = 3, 6, 9, 11, 12, 18); (**e**) DMA-DMAEDM; (**f**) DHMAI.

Many antimicrobial agents that are currently used for domestic or clinical applications are quaternary ammonium salts (QASs), which rely on the cationic charge on the quaternary ammonium group to kill the microorganisms [14]. Due to lacking active functional groups in the chemical structures of QASs, they cannot chemically bond to the dental resin networks, which may result in the burst release of QASs. The antimicrobial functionality of QASs mainly relies on the quaternary ammonium moiety; therefore, modification of the chemical structures on the basis of the original chemical analog to introduce a polymerizable moiety, e.g., C=C, is an effective approach to add active functional groups for polymerization with the establishment of chemical bonds [15,16]. Xiao et al. [17] added a methacryloyl group to a series of QASs to obtain QAMs for dental applications, such as DMAE-BC (Figure 4a), DMAE-m-CBC (Figure 4b), and DMAE-CB (Figure 4c). These QAMs not only maintained the quaternary ammonium structure to realize the antimicrobial properties through cationic charge, but also had acrylic structures to take part in polymerization in the dental resin composite processing. Li et al. [18] synthesized a series of QAMs similar to DMAE-CB but with a chain length varying from 3 to 18 (Figure 4d), and they were introduced to dental adhesives to test the antimicrobial efficacy. Li et al. [19] also synthesized DMA-DMAEDM (Figure 4e) and DMAHDM (Figure 4f) with a chain length of 16 to investigate the effect of quaternary amine charge density on the antibacterial efficacy and bond strength of a dental adhesive. The chemical synthesis of QAMs mainly involves radical polymerization, which is a challenge in the precious control over macromolecular structures, configurations, and their functionalities.

### 2.4. Deep Eutectic Solvent (DES)

Previously, new antibacterial monomers were synthesized through chemical processes, which may couple with problems like by-products, high energy consumption, and high time consumption. Deep eutectic solvent (DES) is a physically prepared eutectic mixture, which has physiochemical properties like ionic liquid (IL) [20,21]. As DES is typically prepared by simply mixing two or more components at a certain ratio at slightly elevated temperature, it is possible to avoid producing by-products and have good economic feasibility [22]. Abbott et al. [23] further divided DES into four types, and the type III DES, which is composed of QAS with a halide anion and a hydrogen bond donor (HBD), was the most attractive. As many antimicrobial agents are QASs, it provides a hint for the use of QAS-based DES in dental resin composite.

**Figure 5:**
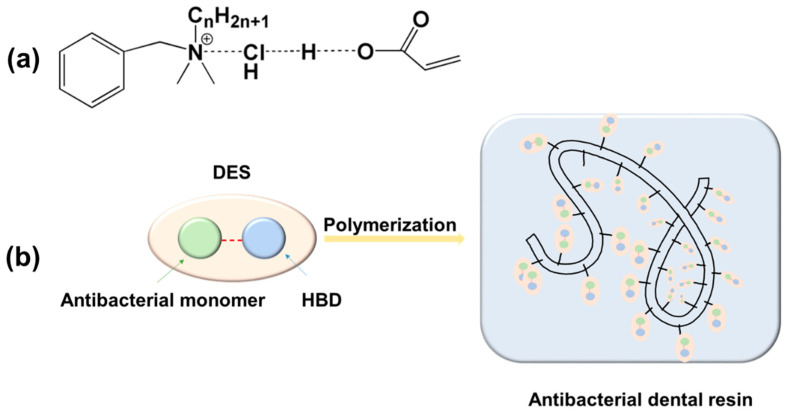
Scheme of deep eutectic solvent (DES): (**a**) Chemical structure of benzalkonium chloride/acrylic acid (BC/AA) DES; (**b**) incorporation of DES into dental resin through polymerization.

Similar to IL, DES is more often used as a solvent for dissolving purposes. Wikene et al. [24] dissolved an antimicrobial agent of curcumin into DES, and they found that a supersaturated solution of curcumin-DES is favorable to lock the confirmation of curcumin, thus enhancing its functionality as a photosensitizer. Compared to IL, DES not only has similar solvent characteristics, but also has advantages in bioactivity modifications through the selection of a single component within the eutectic mixture. Choline chloride-based DESs may not exhibit antimicrobial properties, but when using the same HBD, phosphonium-based DESs may have stronger inhibition effects compared to the single component [25,26]. This indicated that the antimicrobial effect of DES is strongly related to the inhibition behavior of its single component, and a synergistic effect may exist after forming DES. The microbial inhibition effects of ammonium-based DESs [27] and cholinium-based DESs [28,29] further proved that the antimicrobial effects of DES strongly depend on its compositions. Although DES is capable of antimicrobial tasks, it cannot play similar roles to QAMs in a polymer network if it is not polymerizable. In order to have DES covalently bond to dental resin composite networks, a polymerizable group is necessary. DESs prepared by Monta-Morales et al. [30] are mixed eutectics of ammonium salts and HBDs containing acrylic acid (AA) and methacrylic acid (MA); therefore, DESs were able to play an all-in-one role as monomer, filler, and reaction medium. Wang et al. [31] prepared an antimicrobial agent, benzalkonium chloride, and derived polymerizable DES, which was a pioneer work to make DES have the dual functions of bacteria inhibition and polymerization (Figure 5). The antimicrobial and polymerizable DES was further incorporated into dental filling composite [32], which was a relatively new type of antimicrobial monomer for the preparation of dental resins. Unlike the QAMs in the previous part, DES is the physically mixed product of QAS instead of chemically reacted product. Despite the difference in obtaining approaches, DES and QAM have similar properties in both physical properties and chemical reactivity. From this point, DES could be regarded as an unreacted form of QAM synthesis reactant. Because of the elimination of chemical reaction, DES is more simplified than QAM.

## 3. Curing Behavior and Mechanical Properties

Methacrylate resin has been used for a denture base and hardened by thermal curing at the very beginning. Subsequently, resins are cured directly in the mouth, which is the prototype of current dental resin composites practiced in dental clinics [33,34]. The high shrinkage rate of pure monomer methacrylate is the main disadvantage when used directly in the resin, and the degree of conversion (DC%) of methacrylate double bonds is closely related to the mechanical properties of the cured resin composite, which is the key parameter to evaluate the feasibility of applying it to dental restorations [35]. With the development of modern dental composite materials, except for the basic requirement for the instant effect of dental restoration, long-term experience of the restoration, for example, esthetics and wear resistance, is attracting more attention of both dentists and patients [36]. Especially when a new type of functional monomer is introduced to a dental resin composite system that is currently in use, it may significantly affect the curing behavior and the mechanical properties. Main concerns of the curing behavior of dental resin composite focus on the DC%, volume shrinkage, and depth of cure. During the monomer polymerization of composite resin, van der Waals forces are converted into covalent bonds, reducing the distance between monomers and resulting in a volume shrinkage [37]. The mechanical strength of dental filling composite is usually measured by a universal tester for the maximum loads of bending strength, flexural strength, and shear strength of composite resin [38]. Dental adhesives directly contact with dentin or enamel whose mechanical strength is typically evaluated by testing micro-tensile bond strength and shear bond strength [39].

Imazato et al. [40] evaluated the curing behavior of MDPB by incorporating it into a Bis-GMA-based dental composite, and they did not observe an adverse effect after measuring the depth of cure and DC%. They concluded that a small amount of MDPB addition, around 0.5%, was favored to improve the curing properties of the dental resin composite while immobilizing the component in a cured composite. Although color stability of dental composite containing MDPB was affected a little with rapid discoloration, water sorption characteristics could be kept as good as the original recipe [41], which made it possible for clinical applications. When it comes to dental adhesives, the incorporation of MDPB was closely associated with the generations of adhesive development. Eight generations of dental adhesives have been developed, including the most up-to-date universal adhesives until now, and MDPB was practiced for the sixth generation self-etch adhesive, which included two components, a primer and an adhesive [42]. Imazato et al. [43] incorporated MDPB into a proprietary primer within the dental adhesive system at the percentages of 1%, 2%, and 5%. The DC% of the experimental primers with MDPB had no significant difference compared to the control group, and an improvement in bond strength was obtained when adding MDPB at the level of 1% and 2%. They further incorporated MDPB into a proprietary adhesive within the dental adhesive system [44], where no significant differences were observed in curing behavior and tensile bond strength between the MDPB-incorporated group and the control group. Zhang et al. [45] incorporated dual antibacterial agents MDPB and nanoparticles of silver (NAg) incorporated into a dental adhesive, and MDPB + NAg in dental adhesive did not compromise the dentin bond strength. It was demonstrated that the antibacterial potency of MDPB adhesive could be substantially enhanced via NAg because MDPB as contact-inhibition and NAg as long-distance inhibition were complementary to each other. They also combined MDPB and NAg into the primer, which showed that the mechanical properties were not reduced and antibacterial function was also obtained [45]. When combining the MDPB-incorporated primer and MDPB-incorporated adhesive, no adverse effect was introduced with the strengthened resulting antimicrobial effect. Meanwhile, MDPB was the only antimicrobial monomer incorporated into a commercially available adhesive system, Clearfil Protect Bond™ (Kuraray Co. Ltd., Japan) [9,46].

Except for MDPB, other antimicrobial monomers were evaluated on the curing behavior and mechanical properties, but only at experimental sets. Kwon et al. [47] developed a light-curable fluoride varnish containing MPC with expectations to prevent tooth enamel demineralization and improve the anti-biofouling properties, but a decrease in DC% was observed coupled with an unwanted film thickness increase. They found that only a low concentration of MPC, about 3 wt.%, would be effective to reach their targets. Koyama et al. [48] utilized MPC to modify a dental resin composite for controlling plaque formation. The chemical binding of MPC to the surface of resin composite through visible-light irradiation was achievable, and the DC% could reach 55–75%, meeting the requirement. Cao et al. [49] combined MPC and DMAHDM into a dental filling composite, and 3% MPC + 1.5% DMAHDM produced the best protein repellent and antibacterial capabilities without compromising the mechanical properties. Zhang et al. [50] developed a novel resin-modified glass ionomer cement (RMGI) in which MPC was incorporated to reduce protein adsorption for preventing enamel demineralization. The antibacterial properties were achieved without compromising the shear bond strength. They further investigated the effects of MPC on dentine bond strength through mixing MPC with a scotch bond multi-purpose (SBMP) primer, and showed that 7.5 wt.% MPC in the resin had the strongest protein-repellent property without compromising the dentine bond strength [51]. He et al. [52] added a series of QAMs with various lengths of substituted alky chain (from 10 to 18) into Bis-GMA/TEGDMA dental resin, and no significantly adverse impact on double bond conversion and mechanical properties was observed for all cases in their study. Li et al. [53] also investigated the effects of the chain length of QAMs on a dental adhesive, but no significant differences on human dentin micro-tensile bond strength were obtained with varied chain lengths (3, 6, 9, 12, 16, 18, number of CH_2_ groups in QAM analogs). Compared to the control group, they claimed no adverse effect when QAMs with varied chain lengths were added at a mass fraction up to 10%. Xiao et al. [54] put DMAE-CB into dental adhesive and evaluated the micro-tensile bond strength of the resin. The results revealed that the bonding ability of the adhesive was not significantly adversely affected, and a strong and long-lasting bacteriostatic property could be achieved. Antonucci et al. [55] incorporated ionic dimethacrylate monomers (IDMAs), which contain quaternary ammoniums groups, into the Bis-GMA/TEGDMA dental resin system to develop improved biomaterials, and the DC% of the Bis-GMA/TEGDMA dental resin system increased slightly upon incorporation of IDMAs. He et al. [56] added 2-dimethyl-2-dodecyl-1-methacryloxyethyl ammonium iodine (DDMAI) into a Bis-GMA/TEGDMA resin system to investigate the antibacterial and mechanical change in the resin system. The DC% of the polymer had no significant difference, while mechanical properties and the antibacterial effectiveness of the polymer with 3 wt.% DDMAI were higher than those without DDMAI. Li et al. [19] mixed DMAHDM with an alkyl chain length of 16 into an adhesive and primer at different mass fractions, and all groups had similar micro-tensile bond strengths without compromising the dentin bond strength.

As a new type of antimicrobial monomer, studies about the curing behavior and mechanical properties after incorporating DES into dental resins are relatively rare, but a similar trend was addressed that polymers with a successful DC% are more likely to present better mechanical properties. Garcia et al. [57] demonstrated a new orthodontic adhesive with an imidazolium ionic liquid (BMIM. NTf_2_) as the antibacterial agent. The incorporation of BMIM. NTf_2_ had no adverse influence on the DC% of dental adhesive resin. Garcia et al. [58] further evaluated the effect of tantalum oxide quantum dots (Ta_2_O_5_QDs) with an imidazolium ionic liquid as the antibacterial agent for adhesive resin on the DC%. The addition of Ta_2_O_5_QDs to the adhesive resin showed no significant difference from the control (the adhesive without Ta_2_O_5_QDs), and therefore, it did not affect the DC% or the bond strength of dentin. It was indicated in their work that the addition of ionic liquids had no adverse effect on the curing behavior and mechanical properties of resin composite. The application of ionic liquids in the dental resin composite has paved the way for the development of DES in dental resin composites. DES was only incorporated into a dental filling composite in a preliminary work [32]. The flexural strength of the 1.5% DES-incorporated composite could be maintained at a similar level compared to the control group, but the curing behavior was not investigated in the current work. Although no systematic reports were available for these antimicrobial monomers incorporated into dental resin composites, it demonstrated the trend that a relatively small amount of antibacterial agents might be favorable to maintain relatively satisfactory mechanical properties.

## 4. Antimicrobial Properties

### 4.1. Antimicrobial Effectiveness

Different antibacterial agents have different antibacterial action mechanisms against the same pathogenic bacteria, and the same antibacterial agent has different action mechanisms, effectiveness, and inhibition ranges against different pathogenic bacteria, etc. Imazato et al. [59] studied the antimicrobial effectiveness of water-soluble and water-insoluble MDPB-based resin composites, and found that the antimicrobial effectiveness of MDPB polymers was reduced after immobilization, while the latter had little antimicrobial activity. However, a MDPB-curing resin still showed a bacteriostatic effect on oral *streptococci*. In addition, Imazato et al. [44] also investigated the antibacterial effectiveness of an adhesive composite incorporating MDPB. The adhesive system exhibited an adverse effect on the *S. mutans* growth, but the mechanical strength of experimental adhesive resin had no significant change. Imazato et al. [43] further incorporated MDPB into a dentin primer to compare the antibacterial effectiveness with other primers, and the MDPB-based dentin primer produced greater inhibition zones, that is, the incorporation of the antibacterial monomer MDPB into the dentin primer and adhesive system did have an antibacterial effect on bacteria. Similarly, they also found that the growth of all species was inhibited via contact with MDPB [60]. Imazato et al. [9] investigated the antibacterial effect of a single-bottled dentin primer containing 5 wt.% of the antibacterial monomer MDPB, and indicated that inclusion of antibacterial monomer MDPB was effective to provide the dentin primer with reliable bactericidal activities. Kuramoto et al. [61] incorporated the MDPB-containing primer into an adhesive system, and it was found that the progression of root caries in vitro was inhibited effectively through a combination of the antimicrobial activity of the adhesive system and the sealing of the demineralized dentin. The previous reports have shown the antibacterial effect of MDPB in vitro. Imazato et al. [62] also reported that MDPB inhibited bacteria in cavities under in vivo conditions, taking the case of dog teeth as an example; the infected cavities were restored and no bacteria were recovered through the use of MDPB-based primer. Izutani et al. [63] analyzed the antibacterial characteristics of MDPB monomer in detail. It was found that MDPB was lethal to the biofilm of *S. mutans* in a short time period at high concentrations (1000 µg/mL), and MDPB inhibited metabolism of the bacteria at low concentrations (4–8 µg/mL).

QAMs have been made to develop antibacterial resin composites and adhesive systems. Monomers, such as UDMQA, DMAEDM, and DMADDM, had been incorporated by researchers to test the antimicrobial effectiveness [64,65]. Liang et al. [66] synthesized a series of novel urethane dimethacrylate quaternary ammonium methacrylate monomers (UDMQAs) for dental materials, and the UDMQAs could be used to replace Bis-GMA as base monomers of dental resin composites. Compared to the Bis-GMA-based dental resin composite, UDMQA-based resin was successfully endowed with antimicrobial effectiveness. Huang et al. [67] elaborated the antimicrobial effectiveness of the quaternary ammonium dimethacrylate monomer IMQ-16 by incorporating it into the diurethane dimethacrylate (UDMA)/tricyclodecane dimethanol diacrylate (SR833s) resin system. Compared to Bis-GMA/TEGDMA dental resin, IMQ-16 17 wt.% or 20 wt.% can endow the UDMA/SR833s resin system with valid antimicrobial effectiveness. Liang et al. [68] added four novel quaternary ammonium dimethacrylate monomers named IMQ (side alkyl chain length from 12 to 18) into the Bis-GMA/TEGDMA dental resin system with a series of mass ratios, and IMQ-16 in the range of 5 wt.%–10 wt.% was claimed to be the best antibacterial agent. The alkyl chain length of QAMs was considered a significant part in the antimicrobial effectiveness of dental resin composites. Li et al. [53] investigated the antibacterial activity of DMAEDM and DMADDM against eight different species of oral pathogens, and they had strong antimicrobial effectiveness and could rapidly and efficiently be sterilized. DMADDM had a higher bacterial inhibition than DMAEDM, because DMADDM had an alkyl chain of 12 carbons, boosting its antimicrobial activity. He et al. [52] incorporated a series of QAMs with different substituted alkyl chain lengths (from 10–18) into dental resins as antimicrobial agents. They found that the antimicrobial effectiveness of resin composite increased with the substituted alkyl chain length (from 10–18) of QAM. Similarly, He et al. [69] also synthesized a series of QAMs with the aim of using them as immobilized antibacterial agents in methacrylate dental resins, and it was indicated that the antibacterial activity varied parabolically with the alkyl side chain length of these monomers.

QAMs have been synthesized to develop antibacterial resins and adhesives that can kill or restrain bacteria, but the clean dental resin is easily coated with a salivary pellicle comprised of adsorbed salivary proteins, which causes oral bacteria to attach [70,71]. Therefore, it would be desirable to develop a new protein repellency and to inhibit protein adsorption. Cherchali et al. [72] mixed the quaternary ammonium dimethyl-hexadecyl-methacryloxyethyl-ammonium iodide (DHMAI) and the MPC as antimicrobial agents for a methacrylate-based dental resin composite, and investigated the influence of each alone and then their combined effect. It was found that the incorporation of DHMAI in resin can improve antimicrobial effectiveness, while the addition of MPC to DHMAI did not provide a better antimicrobial effectiveness and affected the mechanical properties of composites. Thongthai et al. [73] utilized the MPC polymer possessing the function of protein repellency to address the problem, in which the effectiveness of immobilized bactericide was reduced by salivary protein coverage. Both the MDPB and MPC were able to fabricate a novel copolymer, which served as a surface coating on a methacrylate-based dental resin, but the MDPB/MPC copolymer exhibited a more hydrophilic surface than that provided by MDPB, reducing the adsorption of salivary protein. That is, the resin composite showed promising protein-repelling and bacteria-inhibiting properties. Zhang et al. [13] combined a protein-repellent MPC and antibacterial agent quaternary ammonium DMAHDM into dental resin composite, and investigated the effects of MPC and DMAHDM on resin composite. The composite with 3 wt.% MPC + 1.5 wt.% DMAHDM had better reduction in biofilm growth than that of MPC and DMAHM alone and was promising to inhibiting secondary caries. Zhang et al. [74] further incorporated MPC as a protein repellent into TEGDMA resin composite to inhibit bacteria in the oral cavity. The resin composite with 3 wt.% MPC greatly reduced protein adsorption. Zhang et al. [75] also added 7.5 wt.% MPC into a primer and adhesive to achieve the lowest protein adsorption and greatly reduce the bacterial adhesive.

As investigations about the antimicrobial effectiveness of DES or ionic liquid (IL)-incorporated dental resin composites are in a relatively small number, findings of DES-based or IL-based dental resin are limited. Garcia et al. [58] synthesized Ta_2_O_5_QDs using an imidazolium ionic liquid and appended it to an experimental adhesive resin. The antimicrobial effectiveness against biofilm formation of bacteria was improved, and the adhesive resin formulated might be suitable for restorative purposes. Compared to ionic liquid, the preparation procedure of DES was much greener and easier [76]. Wang et al. [32] incorporated a BC-based DES into a dental resin composite, which provided the polymer with greater antimicrobial effectiveness by controlling the diffusion of the antibacterial agent. The increasing need of antimicrobial resin composites prepared with less complexity and better antimicrobial effectiveness has urged researchers to conduct intensive research in this area. 

### 4.2. Releasing Profile

Cured MDPB-containing primer has an inhibitory effect on the growth of all species, which were in contact with the specimen surface, and displayed little bactericidal effect on *S. mutans* without releasing any unpolymerized antibacterial components [60]. Imazato et al. [8] investigated the inhibited effect of MDPB-containing dentin primer on oral bacteria after curing, indicating that no antibacterial component was eluted from the primer after curing by the agar-disc diffusion method. Similarly, Imazato et al. [7] incorporated MDPB after curing into dental resin composite to study the antibacterial effectiveness and release profile, and no antibacterial components were released from resin composite. The antibacterial activity of MDPB resin in the water-soluble and water-insoluble form was estimated; there was no correlation between the sensitivity of bacteria to unpolymerized and polymerized MDPB; and the difference of antibacterial effectiveness may be due to various adhesion rates [70]. Imazato et al. [77] also incorporated unpolymerized MDPB into dental resin composite to investigate the release profile of the antibacterial component. Although MDPB was eluted at 1 µg/mL from the resin, there was little influence on bacterial growth and could thus be neglected.

**Figure 6:**
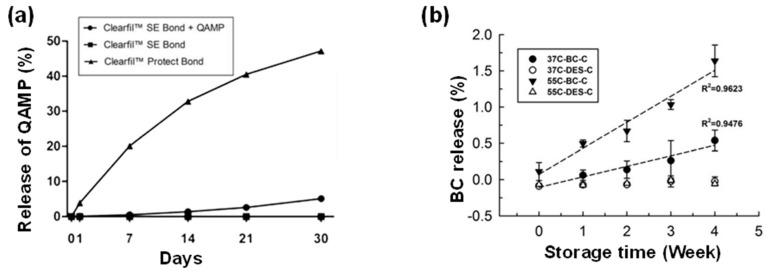
Releasing profile of antimicrobial monomers: (**a**) Release of quaternary ammonium compounds (QAMs) from adhesive systems (adapted from reference [80]); (**b**) release of antibacterial agent BC from dental filling composite systems (adapted from reference [32]).

QAMs as one of the most effective antibacterial agents are widely applied to dental resin composites, and they have less safety concerns and have better esthetic properties. Inorganic fillers do not have chemical linkage to the dental resin, and can easily release over time, which may lead to health risks. QAMs can form chemical bonds with resin composites to improve their bond to dental resin [78,79]. Pupo et al. [80] synthesized a quaternary ammonium methacrylate polymer (QAMP) with antimicrobial potential, and added it into a dental adhesive system. The results indicated that the release of QAMP from resin composite was very low after 30 days. As was demonstrated in Figure 6a, Clearfil™ SE Bond containing 5% QAMP showed an only 5.1% release of quaternary ammonium compounds while Clearfil™ Protect Bond released 47.2% of MDPB after 30 days. QAMP could be considered an innovative antimicrobial approach for the development of new dental adhesive systems. Mirizadeh et al. [81] studied the antibacterial effect of DMAEMA monomer on a denture-base resin in vitro, indicating that DMAEMA in dental resin was not eluted as the agar diffusion test showed, and the resin had a high antimicrobial effectiveness. In addition, Beyth et al. [82] investigated the antibacterial activity of dental composites containing quaternary ammonium polyethylenimine nanoparticles (PEI) against *S. mutans*, and the results indicated that PEI immobilized in dental resin had a strong antibacterial activity upon contact without the release of the nanoparticles. Zhang et al. [75] incorporated a novel QAM based on sol–gel reaction into dental adhesive, and found that QAM could kill bacteria not only via the release of nonpolymerizable quaternary ammonium silane species but also through the effect of immobilized quaternary methacryloxy silane. Xiao et al. [54] incorporated 3 wt.% DMAE-CB into an adhesive and the antibacterial activity of the resin lasted for at least 6 months, which is similar to that of resin composite added with MDPB. The adhesive exerted stable and long-lasting antibacterial effects because this monomer was immobilized in the resin with limited release of bacterial agent. Burujeny et al. [83] evaluated the antibacterial activity of QAMs against *S. aureus* and *E. coli* to judge the release behavior of antibacterial agent in resin composite. It was indicated that no inhibition zone was detected around the samples, and the nonreleasing behavior of active ingredient was confirmed.

DES and QAM have similar properties in terms of physical properties and chemical reactivity, and the antibacterial effect of the DES-based antimicrobial agent is closely related to the inhibition behavior of a single component [84]. Wang et al. [32] evaluated the releasing profile of a dental resin composite with DES composed of AA and BC. It was noted that adding BC-based DES instead of BC alone into dental resin composite could minimize the release of BC, which might be due to hydrogen bonding within AA and BC (Figure 6b). The releasing profile of antimicrobial monomers in dental resin limits the amount of antibacterial agent used in both dentin composite and adhesive resin.

### 4.3. Biocompatibility

The dental resin composite currently in use mainly contains acrylate monomers that convert to acrylic resins after polymerization. Dentists and dental patients are exposed to the monomers during dental treatment and the polymers after dental restorations, which may cause local mucosal irritation or allergic reaction due to their cytotoxicity [85,86]. After chemical structure modifications, antimicrobial properties may be added, which may also enhance the risks of possible complications during the dental procedures, leading to biocompatibility concerns [87]. From this point, the biocompatibility of antimicrobial polymers applied in dental resin composite is important to address, which may correspond to unpredictable issues when a new antimicrobial monomer is added.

Imazato et al. [88] studied the cytotoxicity of antimicrobial monomer MDPB on human pulpal cells, and no cytotoxic effect of MDPB was observed on contact with MDPB at concentrations of 10 µg mL^−1^ or less. It was suggested that MDPB can be effectively incorporated into dental resin composite to provide antibacterial activity against oral bacteria with acceptable biocompatibility. Imazato et al. [89] further incorporated antibacterial monomer MDPB into self-etching primers to investigate the cytotoxic effects of the MDPB-based resin composite on human pulpal cells by in vitro dentine barrier tests. The results showed that the specimens with the MDPB-based primer had a 26%–35% reduction in cell activity. Although toxicity of the MDPB-based dental resin composite was observed, there was no significant influence on the cytotoxicity. MDPB is synthesized by combining a quaternary ammonium compound with a methacrylate group, which is an antibacterial monomer incorporated in the resin system [90]. However, the MDPB-based adhesive may result in inflammation and necrosis of the pulp, which can be attributed to toxic effects of dental resin monomers [91]. The protective effects of N-acetyl cysteine (NAC) against cytotoxicity induced by conventional dental resin monomers have been widely documented [92]. Ma et al. [93] investigated possible protective effects of NAC against the cytotoxicity of MDPB, and the cytotoxicity of MDPB decreased when mixing NAC and MDPB. Ag has been confirmed to possess antibacterial properties, and has a low toxicity and good biocompatibility with human cells [94,95]. It was suggested as beneficial to use a low NAg filler level in the resin [96]. Zhang et al. [45] incorporated MDPB and NAg into dentin primer to investigate fibroblast cytotoxicity and antimicrobial properties, and the results showed no difference in cytotoxicity between commercial control and antibacterial primers. It was indicated that MDPB + NAg may have wide applications in other materials to inhibit bacteria. Zhang et al. [45] further added MDPB and NAg into an adhesive and primer to investigate the effects of combining the MDPB + NAg primer with the MDPB + NAg adhesive. The results suggested that MDPB and NAg as dual antibacterial agents in the adhesive and primer had a multiplying effect in reducing the biofilm activities. Even with higher eluent concentrations, the MDPB and NAg groups still had nearly 100% of fibroblast viability. Therefore, the combination of MDPB + NAg on the adhesives and primers was able to produce strong antibacterial effectiveness without adversely affecting the cytotoxicity.

QAMs were first added into dental resin composites to combat dental caries in the 1990s [64], and some researchers modified QAMs in different ways on the anti-biofilms effects, cytotoxicity, and biocompatibility. Liang et al. [97] synthesized a new QAM, triethylaminododecyl acrylate (TEADDA), and studied the effects of the changed position of the functional groups of TEADDA on the cytotoxicity and biocompatibility of adhesive resin. There was no significant difference in the cytotoxicity and biocompatibility between DMADDM and TEADDA, that is, TEADDA-based adhesive did not cause a significant inflammatory response to dentin, which ensured a good cytotoxicity and biocompatibility. Zhang et al. [75] analyzed the cytotoxicity results from quaternary ammonium species with and without methacryloxy functional groups created by one-pot sol–gel processing. They concluded that the inclusion of antimicrobial component quaternary ammonium species did not adversely affect the cytotoxicity of the dentine adhesive. Burujeny et al. [83] found that the copolymerization of quaternary ammonium salt-containing monomers (QASM) incorporating thiolene-rich resin improved the biocompatibility of resin, which provided significant improvement in the biocompatibility of dental materials. Li et al. [53] incorporated monomers of DMAEDM, DMADDM, and Bis-GMA into dental resin composites, and measured their cytotoxicity using a methyl thiazolyltetrazolium assay and live/dead viability assay. DMAEDM and DMADDM had twenty times less cytotoxicity than Bis-GMA, which was promising for the clinical usage such as adhesive and other restorative materials. Han et al. [98] investigated the effects of dental adhesives containing QAMs with different chain lengths on bacterial properties, and found that the cytotoxicity of adhesives containing QAMs can be tailored by controlling alkyl chain lengths. Zhou et al. [99] demonstrated the effects of the chain length of DMAHDM on the bacterial viability and cytotoxicity, and the eluents from the cured resin containing DMAHDM affecting the fibroblast viability were not significantly different from the resin control without DMAHDM. Li et al. [100] also reported that a new QAM-containing bonding agent can reduce the *S. mutans* biofilm without compromising bond strength and cytotoxicity.

The preparation of new dental biomaterials used in the bonding of prostheses to dentinal tissues require fundamental changes in modern dentistry, and MPC as a new methacrylate also had excellent biocompatibility [101]. Ishihara et al. [102] demonstrated the cytotoxicity of MPC in cells, and the phospholipid polymer MPC could penetrate the cell membrane by molecular diffusion, carrying the bioactive compound inside the cell, without exhibiting significant cytotoxicity. DESs have the advantages of convenient synthesis, lower costs, and lower toxicity, and DESs have been widely applied in the areas of polymer science, nanomaterials synthesis, and drug delivery [27,29]. Wang et al. [32] reported that the BC-based DES-incorporated dental resin composite produced a better biocompatibility than the BC-based composite. It may be that the cytotoxicity and biocompatibility of the DES depended on BC, and the limited release of BC contributed to greater cytotoxicity and biocompatibility of dental resin composite. All in all, the cytotoxicity and biocompatibility of resin play important roles in their clinical success. Further investigations are needed to study the mechanisms and the efficacy of the cytotoxicity and biocompatibility of resin composites to meet requirements of clinical applications.

According to ISO 10993, the biocompatibility of dental resins can focus on cytotoxicity, sensitization, genotoxicity, etc. MDPB, with the methacryloyl group, is usually used as an antibacterial monomer with polymerizable abilities. However, there is some evidence that methacrylate-based composite components may cause sensitivity, although few clinical trials have been conducted. Henriks-Eckerman et al. [103] investigated the sensitization of the methacrylates and determined their concentration in commercial dental restorative materials. Hagberg et al. [104] reported that dental personnel exposed to 2-hydroxyethyl methacrylate and methyl methacrylate may have an irritant effect. They further determined the level at which irritant effects would not be expected in healthy people. Moreover, the volatility of methacrylate can easily lead to respiratory complication and has been associated with adult onset asthma and rhinitis. It was found that skin diseases of dental patients are concerned with the sensitivity of the methacrylate-based dental resin composite. Methacrylates are now ubiquitous in current dental resins and cannot be eliminated. It should be made aware that dental resins contain potent sensitivity, and dental personnel should pay more attention to materials, as well as use them suitably in the workplace to reduce risk [105]. In addition, genotoxic effects of the materials are indicative of the interaction between DNA and antibacterial monomers. For example, Bis-GMA as an antibacterial monomer may damage the DNA of bacteria to some extent, because Bis-GMA tested positive in the DNA synthesis inhibition test, which causes genotoxicity [106]. Besides, the mitochondria of bacteria was damaged, leading to cell death, which was through the induction of apoptosis by the monomer. Lefeurvre et al. [107] found that TEGDMA could induce lipid peroxidation and mitochondrial damage, leading to cell death. Antibacterial monomers are essential for dental pulp repair currently, and we should use them on the basis of weighing the advantages and disadvantages. Therefore, it is important to identify potential adverse effects of dental materials on the health of people.

## 5. Conclusions and Future Perspective

Antibacterial dental resin composites could be synthesized by incorporating antimicrobial agents—MDPB, MPC, QAM, and DES—for dental restorations to combat secondary caries. Each antimicrobial ingredient has specific effects on the mechanical properties and antibacterial properties of dental resin composites. This review has summarized the use of four types of antimicrobial monomers in dental resin, and their effects were demonstrated on the curing behavior, mechanical properties, antimicrobial effectiveness, releasing profile, cytotoxicity, and biocompatibility of resins. The ratios and species of antimicrobial monomers in dental resin composites are important factors to affect the properties of resins. It is also possible to adjust the physiochemical properties of resins by altering antimicrobial monomers to better fit certain applications in the modern clinic. Among these, most of the previous works on the development of antimicrobial dental resin composites are in vitro, and little attention has been paid to them in vivo. Therefore, it will be required to evaluate the clinical application of antimicrobial dental resin composites in vivo. In addition, researchers are suggested to pay more attention to the balance between the antimicrobial properties and mechanical properties of dental resin composites. In-depth research is also necessary to determine whether antibacterial resin composites induce bacterial drug resistance. As far as we know, only one study for dental resin composites incorporated with DES with a controlled releasing profile of antimicrobial monomers or other agents has been reported. The development of DES-based dental resin composites can become a promising approach for the further improvement of dental resin composites. This review aimed to provide a summary of previous works toward antibacterial dental resin composites, and hopefully to offer hints of new ingredients and antimicrobial monomers for future studies corresponding to the antimicrobial properties of dental resin composite.

## Abbreviations

MDPB: Methacryloyloxydodecylpyridinium Bromide; Bis-GMA: 2,2-bis[4-(3-methacryloxy-2-hydroxypropoxy) phenylpropane; TEGDMA: Triethyleneglycoldimethacrylate; DMAEMA: Dimethylaminoethylmethacrylate; PC: Phosphorylcholine; MPC: 2-methacryloyloxyethyl phosphorylcholine; DMAHDM: Quaternary ammonium dimethylaminohexadecyl methacrylate; QAMs: Quaternary ammonium methacrylates; QASs: Quaternary ammonium salts; DMAE-BC: Methacryloxylethyl benzyl dimethyl ammonium chloride; DMAE-m-CBC: Methacryloxylethyl m-chloro benzyl dimethyl ammonium chloride; DMAE-CB: Methacryloxylethyl cetyl ammonium chloride; DES: Deep Eutectic Solvent; IL: Ionic liquid; HBD: Hydrogen bond donor; BC: Benzalkonium chloride; AA: Acrylic acid; MA: Methacrylic acid; Nag: Nanoparticles of silver; RMGI: Resin-modified glass ionomer cement; SBMP: Scotch bond multi-purpose; IDMAs: Ionic dimethacrylate monomers; DDMAI: 2-Dimethyl-2-dodecyl-1-methacryloxyethyl ammonium iodine; Ta_2_O_5_QDs: Tantalum oxide quantum dots; DMAEDM: Dimethylammoniumethyl dimethacrylate; DMADDM: Dimethylaminododecyl methacrylate; UDMQA: Urethane dimethacrylates quaternary ammonium methacrylate; IMQ-16: *N*,*N*-bis[2-(3-(methacryloyloxy)propanamido)-ethyl]-*N*-methylhexadecyl ammonium bromide; UDMA: Diurethane dimethacrylate; SR833: Tricyclodecane dimethanol diacrylate; DHMAI: Dimethyl-hexadecyl-methacryloxyethyl-ammonium iodide; QAMP: Quaternary ammonium methacrylate polymer; PEI: Polyethylenimine nanoparticles; TEADDA: Triethylaminododecyl acrylate; QASM: Quaternary ammonium salt containing monomers; NAC: *N*-acetyl cysteine.
